# Pregnancy and COVID-19: Comparing ICU Outcomes for Pregnant and Nonpregnant Women

**DOI:** 10.3390/v17010051

**Published:** 2024-12-31

**Authors:** Małgorzata Lipińska-Gediga, Waldemar Goździk, Jakub Śmiechowicz, Barbara Adamik

**Affiliations:** Clinical Department of Anesthesiology and Intensive Therapy, Wroclaw Medical University, Borowska 213, 50-556 Wroclaw, Poland; waldemar.gozdzik@umw.edu.pl (W.G.); jakub.smiechowicz@umw.edu.pl (J.Ś.); barbara.adamik@umw.edu.pl (B.A.)

**Keywords:** COVID-19, acute respiratory failure, acute respiratory distress syndrome, organ failure, pregnancy, sepsis, septic shock, ICU treatment

## Abstract

Background: This study compares organ dysfunction, treatment strategies, and unfavorable outcome rates between pregnant and nonpregnant women admitted to the ICU with severe COVID-19, highlighting the increased susceptibility of pregnant women to respiratory infections due to physiological changes. Methods: A retrospective, age-matched study was conducted at a referral center specializing in critical care for pregnant women. Data from 14 pregnant/postpartum and 11 nonpregnant women were analyzed at ICU admission and on days 3, 5, and 7. Results: Acute respiratory distress syndrome was diagnosed in 100% of the pregnant/postpartum group and 64% of the nonpregnant group (*p* = 0.026). Inflammatory parameters were similar between groups, except for lower ferritin levels in the pregnant/postpartum group compared to the nonpregnant (120 vs. 568 µg/L at admission and 90 vs. 616 µg/L on day 3). Creatinine, lactate, and lactate dehydrogenase levels were significantly lower in the pregnant/postpartum group. A reduction in the SOFA score was observed over time in the pregnant/postpartum group (from 7.0 to 4.0 points, *p* = 0.009), while no change was noticed in the nonpregnant group (from 3.0 to 2.5 points, *p* = 0.181). Unfavorable outcome rates were similar, with two patients from each group succumbing to the disease (*p* = 0.604). Conclusions: The findings suggest that pregnancy does not increase the risk of unfavorable outcomes among women with severe COVID-19 receiving ICU treatment. However, additional studies with larger sample sizes are needed to validate these observations.

## 1. Introduction

Pregnancy-specific risk factors for ICU admission include preeclampsia, amniotic fluid embolism, tocolytic-associated pulmonary edema, and peripartum sepsis. Hormone-mediated shifts in type 1/type 2 T-helper cells lead to transient immunosuppression during pregnancy, particularly from weeks 13 to 27 [1]. This immunosuppression is characterized by reduced natural killer cells, T-helper cells, and T-cytotoxic cells, which increase susceptibility to viral and fungal pneumonias [1]. Additional pregnancy-related changes, such as diaphragm elevation, decreased chest wall compliance, reduced lung volume and functional residual capacity, progesterone-induced upper airway hyperemia, and increased oxygen consumption, further predispose pregnant women to respiratory infections caused by intracellular pathogens [2,3]. During the COVID-19 pandemic, ARDS (acute respiratory distress syndrome) emerged as the predominant cause of ICU admissions among pregnant women, surpassing other reasons for ICU care. This condition posed significant life-threatening risks to both the mothers and their fetuses. A recent meta-analysis demonstrated that the most severely ill COVID-19 patients met the Sepsis 3.0 criteria for sepsis/septic shock, with ARDS being the most frequent organ dysfunction [4]. Metz et al. reported that 8% of pregnant women with COVID-19 experienced a severe course of the disease and 4% experienced a critical course [5]. Pregnant patients with COVID-19 were found to have higher rates of ICU admission, mechanical ventilation, and extracorporeal membrane oxygenation (ECMO) use compared to nonpregnant individuals. Furthermore, pregnant patients with critical COVID-19 faced increased risk of preterm birth and venous thromboembolism [2,6,7]. Allotey et al. found that pregnant women with COVID-19 had elevated risks of cesarean section and maternal death compared to their non-COVID-19 counterparts [7]. In contrast to these data, van Genderen et al. reported no cases of death among 26 pregnant women with COVID-19 admitted to the ICU [8].

The objective of this study was to analyze differences in the clinical course of COVID-19 between critically ill pregnant or early postpartum women and nonpregnant women of reproductive age. The primary endpoints were ICU and hospital unfavorable outcome, while secondary endpoints included serious morbidity events such as sepsis, thromboembolism, and acute kidney injury. Additionally, the frequency of ICU admissions among pregnant women during the studied period was compared with that of previous years.

## 2. Materials and Methods

The presented results come from the Clinical Department of Anesthesiology and Intensive Care of the Medical University, which was the referral center admitting pregnant/postpartum women in critical condition due to severe respiratory failure related to COVID-19. We conducted a retrospective, single-center age-matched study comparing pregnant or postpartum patients with women of reproductive age treated at the Intensive Care Unit (ICU) between October 2020 and March 2022. The study protocol was accepted by the Bioethical Committee of Medical University (No. 394/2021 and No 214/2023) and the study was conducted in accordance with the Helsinki Declaration of 1975, as revised in 2013. Informed consent was waived by the Bioethical Committee due to the retrospective nature of this study. All data for statistical analysis were collected from hospital medical records.

### 2.1. Patients

The inclusion criteria for the study were as follows: confirmed SARS-CoV-2 (severe acute respiratory syndrome coronavirus 2) infection, acute respiratory failure due to SARS-CoV-2 infection, female gender, and pregnancy/postpartum (study group only). Exclusion criteria were as follows: cause of acute respiratory failure other than SARS-CoV-2 infection, male gender, and age over 45 years. ICU female patients diagnosed with SARS-CoV-2 who met the inclusion/exclusion criteria were retrospectively divided into two groups depending on whether they were pregnant/postpartum women (study group) or nonpregnant women in reproductive age (control group). Demographic data, concomitant illnesses, clinical course, and obstetric, perinatal, and maternal data were retrieved from the hospital medical records by the physicians involved in this study. For each patient, data recorded at four time points were analyzed: T1 (the day of admission to the ICU), T2 (day 3 of ICU treatment), T3 (day 5 of ICU treatment), and T4 (day 7 of ICU treatment). In addition, data recorded prior to ICU admission were analyzed.

### 2.2. Diagnosis and Management

The basic principles of diagnosis and treatment of COVID-19 were the same for pregnant/postpartum and nonpregnant patients. SARS-CoV-2 infection was diagnosed by positive polymerase chain reaction (PCR) from nasopharyngeal swabs. A diagnosis of sepsis and septic shock was based on the Sepsis-3 definition [9]. Pneumonia was confirmed by X-ray or computed tomography scanning and characterized by diffuse, bilateral ground-glass opacities. Acute respiratory distress syndrome (ARDS) and the applicable stage were determined according to the Berlin ARDS definition [10]. Acute kidney injury (AKI) was diagnosed according to the Kidney Disease: Improving Global Outcome (KDIGO) guidelines [11]. Two clinical scores were used to assess the clinical status and the degree of organ dysfunction, such as the Acute Physiology and Chronic Health Evaluation (APACHE II) [12] calculated upon admission to the ICU and the Sequential Organ Failure Assessment (SOFA) [13], calculated daily.

To support lung function, high-flow nasal oxygen therapy (HFNO) or mechanical ventilation was applied. If indicated, veno-venous extracorporeal membrane oxygenation (V-V ECMO) was administered for respiratory support. The indications for V-V ECMO during pregnancy were the same as in the general population: PaO_2_/FiO_2_ < 50 mmHg for >3 h or PaO_2_/FiO_2_ < 80 mmHg for >6 h; pH < 7.25 with PaCO_2_ > 60 mmHg for >6 h with a respiratory rate > 35 breaths/min, along with plateau pressure > 30 cm H_2_O [14].

### 2.3. Statistical Methods

Continuous variables are presented as medians with the 25th and 75th percentiles, while categorical variables are summarized as counts and proportions. The distribution of the variables was not normal based on a Shapiro–Wilk test. A Friedman ANOVA was used to analyze changes within groups in performance overtime. Numerical variables were analyzed using the Mann–Whitney U test to assess differences between the group of pregnant and nonpregnant women at four time points (on the day of admission to the ICU and on the 3rd, 5th, and 7th day of treatment). Categorical variables were analyzed using contingency tables and a chi-squared test. Significance was assumed if the probability of the null hypothesis was less than 5% (*p* ≤ 0.05). All analyses were performed using Statistica v.13.3 (TIBCO Software Inc., Palo Alto, CA, USA) under license held by the Wroclaw Medical University.

## 3. Results

**Figure 1:**
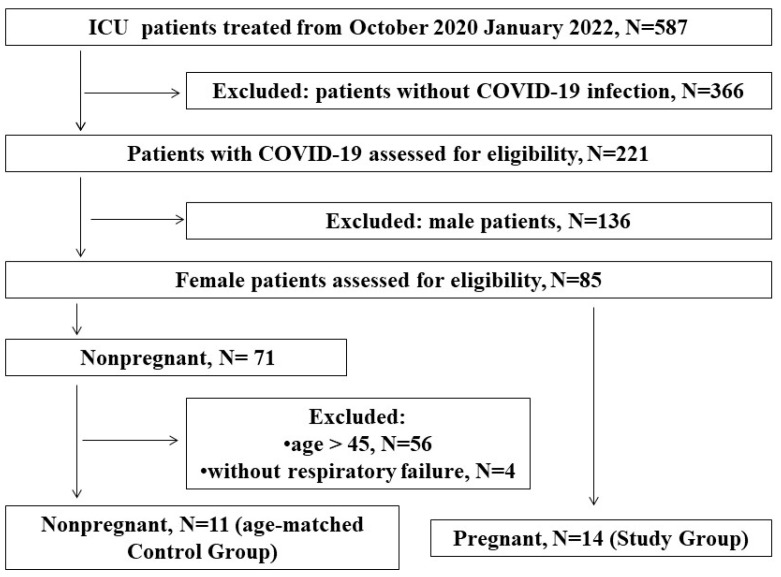
Flow diagram of the study.

Among 587 patients admitted to the ICU between October 2020 and March 2022 (16 months), 221 patients tested positive for COVID-19. SARS-CoV2 virus was identified through an RT-PCR assay of nasal or pharyngeal swab probes in all patients before ICU admission. Among the infected individuals, we identified all pregnant or postpartum women, and all of them were included in the study group (N = 14). We compared this group with a control group comprising nonpregnant women (N = 11) of reproductive age (<45 years, age-matched control) who were treated in the same ICU during the study period and met the inclusion/exclusion criteria. All analyzed patients were diagnosed with sepsis upon ICU admission, and three of them developed septic shock. Figure 1 is a flow diagram of the study participants.

The baseline characteristics of the pregnant and nonpregnant women are summarized in Table 1. The median age of the pregnant patients was 33 years (range: 26–44 years), and the median age of the nonpregnant patients was 39 years (range: 21–45). With the exception of one individual, none of the patients had been vaccinated. All patients were diagnosed with acute respiratory failure upon ICU admission and 84% met the criteria for acute respiratory distress syndrome (ARDS). Patients were followed until hospital discharge or death. The hospital mortality rate in the analyzed cohort was 20% with two patients in the study group and three patients in the control group succumbing to severe respiratory and circulatory failure caused by thromboembolic events. For context, during the same period (October 2020 to January 2022), the overall mortality rate among all hospitalized COVID-19 patients (N = 4381) was 7% (N = 323), whereas the mortality rate among all ICU-admitted COVID-19 patients (N = 587) was significantly higher at 35% (*p* < 0.001).

### 3.1. Clinical Condition of the Study and Control Groups Analyzed Prior to ICU Admission

The majority of patients in the analyzed cohort were admitted to the ICU within the first day of hospital admission. Among pregnant patients, the median duration of hospitalization before ICU admission was 19 h (IQR 1, 69) and 13 h (IQR 1, 116) among nonpregnant patients. All patients (pregnant and nonpregnant) were diagnosed with severe respiratory failure on hospital admission, and 80% met the diagnostic criteria for ARDS indicating a high risk of ICU admission and high risk of mortality. In addition, one pregnant patient and one nonpregnant patient were diagnosed with septic shock. Upon hospital admission, 92% of patients required respiratory support: 4 with an oxygen mask, 11 with high-flow nasal oxygen therapy, and 8 were intubated and placed on mechanical ventilation. Patient management prior to ICU admission, including respiratory support, vasopressor use, and CRRT, did not differ significantly between pregnant and nonpregnant groups (*p* > 0.05) (Table 2). Laboratory parameters measured before ICU admission showed no significant differences between groups, except for CRP, creatinine, and LDH levels, which were lower in pregnant patients compared to nonpregnant patients. Concomitant illnesses are listed in Table 1.

### 3.2. A Comparison of Admission Rates for Pregnant Patients

In order to compare the frequency of ICU admissions of pregnant women in the analyzed period with earlier ICU admissions, an analysis of admissions to the ICU in previous analogous periods was performed. During the study period (October 2020–March 2022), pregnant women requiring ICU treatment accounted for 2.39% of all patients treated in the ICU (14/587). In previous years, admission rates for pregnant patients were significantly lower compared to the admission rate in the period under study; i.e., from October 2018 to March 2020, 628 patients were treated in the ICU, of which 0.38% were pregnant patients (2/628), (*p* = 0.001), and from October 2016 to March 2018, 612 patients were treated in the ICU, of which 0.81% were pregnant patients (5/612) (*p* = 0.028).

### 3.3. Treatment in the ICU

Patient management in the ICU including respiratory support, vasopressors use, and CRRT was similar in pregnant and nonpregnant patients (*p* > 0.05) (Table 3). On admission to the ICU, all patients required respiratory support: 13 with high-flow nasal oxygen therapy and 12 with mechanical ventilation. Vasopressors were administered to maintain mean arterial pressure in 10 patients (40%) and septic shock was identified in 3 patients (1 in the pregnant group and 2 in the nonpregnant group). Continuous renal replacement therapy was administered in 4 patients (1 in the pregnant group and 3 in the nonpregnant group). All patients were administered intravenous dexamethasone at a dose of 6 mg per day. During ICU treatment, extracorporeal membrane oxygenation (ECMO) was administered to eight patients. Six out of fourteen (43%) in the pregnant group and five out of eleven (45%) in the nonpregnant group of patients were prone positioned along with chest and hip support to reduce abdominal pressure.

### 3.4. Inflammatory Parameters

To assess whether there were differences in the intensity of the inflammatory reaction between pregnant and nonpregnant patients, the concentrations of selected biomarkers were compared. The levels of C-reactive protein, WBC, and procalcitonin did not differ between groups (Table 4). Median ferritin levels were significantly lower in pregnant than in nonpregnant patients at T1 (120 vs. 568 µg/L, *p* = 0.032) and at T2 (90 vs. 616 µg/L, *p* = 0.013), with no statistically significant difference at T3 (318 vs. 258 µg/L, *p* = 0.609) (Table 4). At T4, some ferritin measurements were missing, so the analysis from that time point is not shown in the table.

Additionally, we compared the rates of co-infections (bacterial or fungal) found in pregnant and nonpregnant COVID-19 patients on the day of admission to the ICU. Co-infection was confirmed in 36% of pregnant and in 45% of nonpregnant patients. The following pathogens were identified: *Acinetobacter baumannii* (2 patients), *Enterococcus faecium* (2 patients), *Klebsiella pneumonia* (1), *Proteus mirabilis* (1), *Enterobacter cloacae* (1), and *Candida glabrata* (1). Among patients with bacterial co-infections, the median PCT level was significantly higher than in those with an isolated COVID-19 infection (2.01, 1.78, 0.88, 0.51 ng/mL vs. 0.30, 0.22, 0.12, 0.08 ng/mL, at T1, T2, T3, and T4, respectively (*p* < 0.05)).

### 3.5. Organ Failure

**Figure 2:**
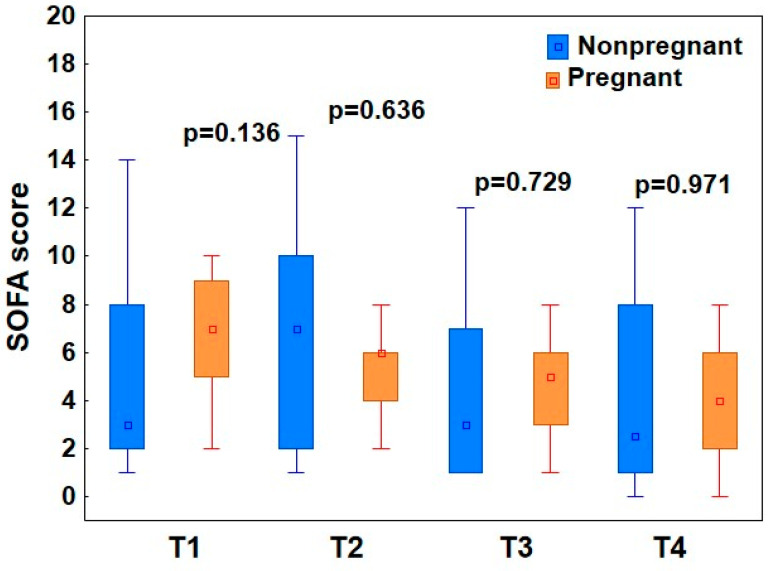
Sequential Organ Failure Assessment score in pregnant and nonpregnant patients. SOFA scores were compared between pregnant and nonpregnant COVID-19 patients. The box plots represent the median values (midpoint) with interquartile range between the 25th and 75th percentiles (box); the whiskers represent the minimum and maximum values.

The SOFA (Sequential Organ Failure Assessment) score was used to assess the patient’s clinical condition on a daily basis, with 0 points being the lowest score (no organ failure) and 24 points being the highest score. There were no significant differences in the SOFA score between the groups at any time point of the study (*p* > 0.05, the Mann–Whitney test) (Figure 2).

Interestingly, when analyzing changes in the SOFA score over time in the group of pregnant patients, the elevated score on ICU admission (7.0, IQR 5, 9) significantly decreased during treatment to the level of 6.0 (IQR 4, 6), 5.0 (IQR 3, 6), and 4.0 (IQR 2, 6) at T2, T3, and T4, respectively (*p* = 0.034, Friedman’s ANOVA). A similar trend was not seen in the group of nonpregnant patients, where the elevated SOFA score on admission (3.0, IQR 2, 8) did not change significantly at T2 (7.0, IQR 2,10), T3 (3, IQR 1, 7), and T4 (2.5, IQR 1, 8) (*p* = 0.181).

The median platelet count was within the reference range in pregnant (236 (IQR 169, 259), 254 (IQR 200, 349), 346 (IQR 263, 382), 359 (IQR 237, 418) × 10^9^/L, and nonpregnant patients (296 (IQR 275, 394), 295 (IQR 216, 390), 284 (IQR 221, 478), 298 (IQR 175, 462) at the T1, T2, T3, and T4 time points, respectively. All patients had an increased D-dimer concentration, i.e., above 0.50 mg/L, which may indicate coagulation disorders (Table 5). The median value of the PaO_2_/FiO_2_ index during the entire time of observation was below 200 in both pregnant (141 (IQR 81, 184), 157 (IQR 100, 231), 122 (IQR 95, 209), 145 (IQR 94, 213) mmHg, and nonpregnant patients (104 (IQR 77, 219), 117 (IQR 103, 340), 140 (127–407), 176 (IQR 113, 319) mmHg at the T1, T2, T3, and T4 time points, respectively, which indicates persistent, severe respiratory failure. The second most common organ failure, after respiratory failure, was circulatory failure in 5/14 (36%) pregnant and 5/11 (45%) in nonpregnant patients, followed by renal failure requiring continuous renal replacement therapy (CRRT) in 1/14 (7%) pregnant and 3/11 (27%) nonpregnant patients. The median levels of creatinine were normal in pregnant and nonpregnant patients, with a significant difference between groups on the day of ICU admission (0.5 vs. 0.9 mg/dL, *p* = 0.019). The median level of lactate was significantly higher in nonpregnant than in pregnant patients only on ICU admission (2.1 vs. 1.3 mmol/L, *p* = 0.002) indicating disturbances in blood perfusion. Septic shock on ICU admission was diagnosed in 1/14 (7%) pregnant and in 2/11 (18%) nonpregnant patients. As an increase in LDH activity may be due to cell damage as well as impaired blood flow and oxygen delivery, we measured LDH activity and found that the median LDH values were above the reference range of 333 U/L in both groups and, moreover, were significantly higher in nonpregnant than in pregnant patients (840 vs. 479 U/L, *p* = 0.026) on ICU admission. At the time point 4, some LDH measurements were missing, so the analysis from that day is not shown in the table.

### 3.6. Pregnancy and Neonatal Outcomes

Eleven out of fourteen women (79%) gave birth during hospitalization, of which seven deliveries were on the day of ICU admission (postpartum patients admitted directly from the operating room after caesarean section) and four during the ICU stay; three women were discharged from the hospital after treatment and gave birth in another medical facility. All women who gave birth while in the ICU had to undergo caesarean section due to the critical condition of the mother. Of the 11 deliveries, 8 were premature, defined as occurring before 37 weeks of gestation, and 3 were at term. All but two of the newborns survived. The cause of death of the newborns was intrauterine asphyxia. Data regarding the outcome of pregnancy is presented in Table 6.

## 4. Discussion

Data from studies on viral respiratory illnesses suggest that infection during pregnancy may worsen both maternal and fetal outcomes [15]. During the study period, pregnant women requiring ICU treatment accounted for 2.39% of all ICU admissions, a marked increase compared to previous years when admission rates for pregnant patients were significantly lower. This study examined differences in the clinical course of COVID-19 between critically ill pregnant/early postpartum patients and nonpregnant women of reproductive age. The primary endpoint was ICU and hospital unfavorable outcome, while secondary endpoints included serious morbidity events such as thromboembolic complications, sepsis, and acute kidney injury requiring renal replacement therapy. The main finding of this study is that, in the analyzed cohort, the clinical course and outcome of pregnant women were comparable to those of nonpregnant women treated in the ICU. However, it is important to note that the sample size was relatively small, and these findings require validation in larger cohorts.

Pregnancy-related physiological changes may predispose pregnant women to a more severe course of COVID-19–related acute respiratory failure compared to their nonpregnant counterparts. Indeed, in our study, ARDS was diagnosed in 100% of pregnant patients and in 64% of nonpregnant patients. Moreover, both groups exhibited persistently low PaO2/FiO_2_ ratios throughout the observation period. Importantly, pregnancy is not a contraindication to any respiratory therapies, and the criteria for administering these therapies are the same as those applied to the general population [16]. In our study, pregnant patients were more likely to receive V-V ECMO support compared to nonpregnant women, reflecting a greater need for advanced respiratory therapy among pregnant patients. Increasing reports highlight the use for ECMO in pregnant patients with COVID-19, with variable treatment outcomes for the mother–fetal dyad [17,18]. According to the ELSO COVID-19 registry, pregnant women accounted for 4% of all reported cases of ECMO for the treatment of COVID-19; however, mortality data for this population were not provided in the registry [19]. In the German multicenter registry of pregnant women infected with SARS-CoV-2 (CRONOS, COVID-19 Related Obstetric and Neonatal Outcome Study), 15% of ICU patients were treated with ECMO in combination with other respiratory support, with an ICU mortality rate of 5% observed in groups treated with various types of respiratory support, including ECMO [20]. Our findings on mortality among pregnant patients requiring ICU treatment and ECMO are similar and suggest that, when clinically indicated, ECMO therapy should be considered for the treatment of COVID-19-related respiratory failure in pregnant and postpartum patients. [21]. Palella et al. conducted a systematic review involving 306 women, of whom 203 (66.3%) were prepartum and 103 (33.7%) were postpartum at the time of cannulation. The results indicated that VV-ECMO in this population could potentially save five out of six mothers (survival > 80%), while fetal mortality rates were doubled, with approximately one-third experiencing unfavorable outcomes (fetal survival rate approximately 67.9%) [22]. In our study, the newborns survival was 86%.

Studies conducted in the United States indicate that pregnant women are 5.4 times more likely to be hospitalized, 1.5 times more likely to require ICU admission, and 1.7 times more likely to need mechanical ventilation compared to nonpregnant women. At the same time, no significant differences in mortality rates were observed between pregnant and nonpregnant women [23]. In our study, the hospital mortality rate in the analyzed cohort was 20%, with two patients in the study group and three patients in the control group succumbing to severe respiratory and circulatory failure. For context, during the same period, the overall mortality rate among all hospitalized COVID-19 patients was 7%, while the mortality rate among all ICU-admitted COVID-19 patients was significantly higher at 35% (*p* < 0.001). These results suggest that pregnant and postpartum COVID-19 patients represent a higher-risk group for ICU admission and unfavorable outcome compared to the general cohort of hospitalized COVID-19 patients. Despite increased risk of severe COVID-19 during pregnancy, vaccine hesitancy has been observed among pregnant women. A recent analysis in six European countries revealed that nearly all pregnant women admitted to the ICU with COVID-19 in the latter half of 2021 were unvaccinated, despite the widespread availability of vaccines [24]. Similar findings were observed in our study, where 100% of pregnant women were unvaccinated. According to Martinez-Varea et al., when comparing maternal outcomes between vaccinated and nonvaccinated pregnant patients with COVID-19, vaccinated women demonstrated a significantly reduced incidence of pneumonia, hospitalization due to COVID-19 symptoms, unfavorable maternal outcomes, and the need for antibiotics, corticosteroids, and oxygen therapy. Notably, pregnant women with SARS-CoV-2 infection who had received at least two doses of the vaccine did not develop severe COVID-19 [25]. Pregnant women with a COVID-19 diagnosis have higher risks of adverse pregnancy outcomes. In the Raffetti et al. study, COVID-19 diagnosis was associated with a higher risk of gestational diabetes (adjusted HR 1.22, 95% CI 1.18–1.26), gestational hypertension (1.16, 1.10–1.22), pre-eclampsia (1.20, 1.12–1.28), preterm birth (1.63, 1.57–1.69, and 1.68, 1.61–1.75 for spontaneous preterm), very preterm birth (2.04, 1.86–2.23), small for gestational age (1.12, 1.07–1.18), thrombotic venous events (1.85, 1.56–2.20) and stillbirth (only within 14 days since COVID-19 diagnosis, 3.39, 2.23–5.15) [26]. Moreover, according to Smith et al., pregnant patients with symptomatic COVID-19 before 20 weeks’ gestation had no increased risk of preterm delivery compared to those testing negative, with adjusted risks of 10.0% (95% CI 7.8, 12.0) vs. 9.8% (9.1, 10.5). Mild COVID-19 later in pregnancy was not clearly associated with preterm delivery. In contrast, severe COVID-19 after 20 weeks’ gestation led to an increase in preterm delivery, and the risk ratio for preterm delivery comparing severe to mild/moderate COVID-19 at 35 weeks was 2.8 (2.0, 4.0); corresponding risk ratios for indicated and spontaneous preterm delivery were 3.7 (2.0, 7.0) and 2.3 (1.2, 3.9), respectively [27]. In our study 11 (79%) out of 14 COVID-19 pregnant women underwent cesarean section and 21% of them were pregnant discharged home.

Ferritin is an acute-phase protein produced by various cells, including cytokine-stimulated macrophages, hepatocytes, and Kupffer cells, during COVID-19 infection [28]. Previous studies have shown that serum ferritin levels increase with the worsening clinical condition of COVID-19 patient [29,30]. An uncontrolled immune response during COVID-19, associated with high ferritin levels and coagulation activation, may lead to multiple organ failure [31]. In our study, ferritin levels at ICU admission were significantly lower in pregnant patients compared to nonpregnant patients, suggesting a less advanced inflammatory response in the pregnant group. This finding aligns with the results of Lin et al., who identified ferritin levels at admission as an independent risk factor for disease severity in COVID-19 patients [30].

Interestingly, when analyzing changes in procalcitonin (PCT) levels over time, we observed that in the group of pregnant patients, the elevated PCT levels at ICU admission significantly decreased during treatment. In contrast, no similar trend was observed in the group of nonpregnant patients, where PCT levels did not significantly change throughout the treatment. Additionally, upon ICU admission, we noted a tendency toward a lower rate of co-infection in the study group compared to the control group. These findings suggest that the study group experienced a less advanced inflammatory process, leading to a more rapid response to therapy. The lack of a significant difference in CRP and PCT levels between the study group and the control group may be attributed to the relatively small sample size.

In this study, the SOFA score in the pregnant group showed a significant decrease during ICU treatment, a trend not observed in the control group. In addition, elevated D-dimers were detected in all patients, indicating coagulation disturbances. Thrombosis may be more prevalent due to the combined pathophysiological alterations in hemostasis associated with both pregnancy and COVID-19 [31]. In our study, this complication led to two fatalities in the pregnant group. Both events exhibited a sudden onset and their thromboembolic nature was confirmed during resuscitation through ultrasonography (USG) imaging, in accordance with European Resuscitation Council (ERC) guidelines [32]. According to Beers et al., during pregnancy, angiotensin II, progesterone, and elevated nitric oxide levels reduce vascular resistance and increase renal plasma flow [33]. Increase in the glomerular filtration rate (GFR) and overall hypervolemia leads to a decrease in serum creatinine levels, which are typically lower in pregnant women compared to pre-pregnancy levels. In our study, mean creatinine levels were lower in pregnant patients than in the control group, with a significant difference on the day of ICU admission. Moreover, only 7% of pregnant patients required CRRT compared to 27% in the control group. This observation may be attributed to the increase in GFR and renal plasma flow, which could contribute to a lower incidence of renal injury in pregnant women.

In early pregnancy, steroids are used to treat conditions such as recurrent miscarriage and fetal abnormalities, including congenital adrenal hyperplasia. In mid- to late pregnancy, antenatal corticosteroids are routinely administered to women at risk of preterm birth, with strong evidence supporting their role in improving neonatal outcomes through fetal pulmonary maturation [34]. Corticosteroids are also a standard treatment for severe COVID-19 in both pregnant and nonpregnant patients. Dexamethasone, demonstrated to be beneficial in the RECOVERY trial, reduced mortality by 2.8% (22.9% vs. 25.7%) and was most effective in patients receiving invasive mechanical ventilation, where mortality rates were 29.3% for dexamethasone treatment versus 41.4% for usual care. This dose has been shown to be non-teratogenic, and both pregnant and breastfeeding patients were included in the trial [35]. Dexamethasone decreases the ratio of angiopoietin-2 to angiopoietin-1, thereby promoting endothelial stabilization and reducing endothelial cell apoptosis, which is critical in reducing mortality in critically ill COVID-19 patients [36]. In our study, pregnant ICU patients with COVID-19 exhibited more severe respiratory failure prior to ICU admission but had significantly lower markers of inflammation (CRP) and organ dysfunction (creatinine, LDH) compared to nonpregnant patients. These differences, observed before the initiation of dexamethasone treatment, suggest a less advanced inflammatory process in pregnant patients, potentially contributing to a more favorable response to dexamethasone immunomodulatory therapy in this group. This is further supported by the significant decrease in SOFA scores observed in the study group, in contrast to the outcomes in the control group.

Lactate dehydrogenase (LDH) is an enzyme whose levels can increase due to cell damage, impaired blood flow, and reduced oxygen delivery [33]. At ICU admission, LDH and lactate concentrations were significantly lower in pregnant women compared to the control group, suggesting less advanced blood perfusion disorders in the pregnant cohort [37]. These findings may indicate a more limited inflammatory response in pregnant patients, potentially linked to physiological immunosuppression, which could facilitate a faster response to treatment and a trend toward shorter ICU and hospital stays. Supporting this hypothesis, Ramanathan et al. reported that outcomes for pregnant and peripartum patients supported on ECMO are comparable to, or better than, those of other patient cohorts [38].

However, there are limitations of this study. As a retrospective, single-center analysis, it is constrained by the small sample size of pregnant or postpartum women and the age-matched control group. Additionally, to ensure comparability between pregnant and nonpregnant patients with critical COVID-19, we did not compare ICU-required patients with non-ICU patients.

## 5. Conclusions

The findings suggest that pregnancy does not increase the risk of unfavorable outcomes among women with severe COVID-19 receiving ICU treatment. However, additional studies with larger sample sizes are needed to validate these observations.

## Figures and Tables

**Table 1:** Baseline patient characteristics on ICU admission.

	Pregnant, N = 14	Nonpregnant, N = 11	*p*-Value
**Age (years)**	33 (32, 38)	39 (29, 43)	0.365
**APACHE II score**	8 (5, 10)	11 (5, 18)	0.135
**SOFA score**	7 (5, 9)	3 (2, 8)	0.143
**ARDS, N (%)**	14(100)	7 (64)	0.026
**PaO_2_/FiO_2_ (mm Hg), N (%):**			0.691
≤100	6 (43)	4 (36)	
101–200	6 (43)	4 (36)	
201–300	2 (14)	2 (18)	
>300	0	1 (9)	
**Septic shock, N (%)**	1 (7)	2 (18)	0.406
**Vaccinated, N (%)**	0 (0)	1 (9)	0.440
**Concomitant illnesses, N (%):**			0.308
autoimmune disease	1 (7)	1 (9)	
hypothyroidism	2 (14)	0	
mitral valve insufficiency	1 (7)	0	
asthma	0	1 (9)	
obesity	2 (14)	3 (27)	
chronic kidneys disease	0	1 (9)	
depressive disorder	0	1 (9)	
**LOS, ICU, (days)**	13 (6, 33)	12 (7, 21)	0.826
**LOS, hospital, (days)**	20 (12, 42)	26 (15, 45)	0.622
**Mortality, ICU, N (%)**	2 (14)	2 (18)	0.604
**Mortality, hospital, N (%)**	2 (14)	3 (27)	0.378

APACHE II, Acute Physiology and Chronic Health Evaluation II; SOFA, Sequential Organ Failure Assessment; ICU, intensive care unit; LOS, length of stay. Continuous variables are presented as medians (25th, 75th percentiles), categorical variables are summarized as counts and fractions. The *p*-value represents differences between the groups.

**Table 2:** A comparison of study groups prior to ICU admission.

	Pregnant, N = 14	Nonpregnant, N = 11	*p*-Value
**ARDS, N (%)**	13 (93)	7 (64)	0.065
**PaO_2_/FiO_2_ (mm Hg), N (%):**			0.034
≤100	6 (21)	4 (36)	
101–200	5 (36)	3 (27)	
201–300	5 (36)	0	
>300	1 (7)	4 (36)	
**Septic shock, N (%)**	1 (7)	1 (9)	0.696
**Respiratory support, N (%):**			0.351
no support	0	2 (18)	
oxygen mask	3 (21)	1 (9)	
high-flow nasal oxygen therapy	6 (43)	5 (45)	
mechanical ventilation	5 (36)	3 (27)	
**Vasopressors, N (%)**	3 (21)	4 (36)	0.351
**CRRT, N (%)**	0	0	-
**Laboratory parameters**			
Procalcitonin, (ng/mL)	0.30 (0.10, 0.80)	0.23 (0.08, 0.80)	0.661
CRP, (mg/L)	106 (102, 109)	115 (112, 118)	<0.001
Lactate, mmol/L	1.25 (0.90, 1.70)	1.5 (1.1, 2.9)	0.216
Hb g/dL	9.7 (8.3, 11.3)	10.7 (8.6, 11.6)	0.366
WBC, (10^3^/uL)	12 (7, 16)	13 (6, 25)	0.978
PLT, (10^9^/L)	201 (134, 243)	240 (214, 402)	0.035
D-dimers, (mg/L)	1.28 (0.97, 1.98)	3.2 (1.26, 16.03)	0.100
Creatinine, (mg/dL)	0.56 (0.53, 0.63)	0.84 (0.60, 1.80)	0.043
LDH, (U/L)	338 (262, 471)	833 (501, 955)	0.002
**LOS prior to ICU, (days)**	1 (1, 3)	1 (1, 5)	0.823

CRP, c-reactive protein; WBC, white blood cells; PLT, platelet count; Hb, hemoglobin; LDH, lactate dehydrogenase ICU, intensive care unit; LOS, length of stay. Continuous variables are presented as medians (25th, 75th percentiles), categorical variables are summarized as counts and fractions. The *p*-value represents differences between the groups.

**Table 3:** Patient management in the ICU.

	Pregnant	Nonpregnant	*p*-Value
**Respiratory support, N (%):**			0.570
high-flow nasal oxygen therapy	7 (50)	6 (55)	
mechanical ventilation	7 (50)	5 (45)	
**Vasopressors, N (%)**	5 (36)	5 (45)	0.466
**CRRT, N (%)**	1 (7)	3 (27)	0.208
**Steroid, N (%)**	14 (100)	11 (100)	-
**V-V ECMO, N (%)**	5 (36)	3 (27)	0.495
**Duration of ECMO, (days)**	16 (13, 17)	19 (3, 60)	0.551

CRRT, continuous renal replacement therapy; ECMO, extracorporeal membrane oxygenation. Continuous variables are presented as medians (25th, 75th percentiles), categorical variables are summarized as counts and fractions. For categorical variables, the *p*-value represents the frequency distribution of the variables in groups. For continuous variables, the *p*-value represents differences between the groups.

**Table 4:** Inflammatory parameters in pregnant and nonpregnant patients recorded at four time points: T1 (the day of admission to the ICU), T2 (day 3 of ICU treatment), T3 (day 5 of ICU treatment), and T4 (day 7 of ICU treatment).

	CRP, (mg/L)		Procalcitonin, (ng/mL)		WBC, (10^3^/uL)		Ferritin, (µg/L)	
	Pregnant	Nonpregnant	Pregnant	Nonpregnant	Pregnant	Nonpregnant	Pregnant	Nonpregnant
**T1**	122	75	0.35	0.20	11	13	120 *	568
	(95, 159)	(55, 141)	(0.20, 1.89)	(0.06, 0.94)	(10, 13)	(6, 17)	(100, 326)	(381, 604)
**T2**	112	73	0.34	0.40	10	14	90 *	616
	(78, 144)	(4, 141)	(0.19, 1.68)	(0.11, 1.47)	(8, 13)	(8, 15)	(57, 147)	(380, 700)
**T3**	74	44	0.21	0.04	9	13	318	258
	(52, 136)	(25, 92)	(0.10, 0.56)	(0.12, 0.65)	(8, 10)	(9, 18)	(73, 430)	(189, 1209)
**T4**	75	51	0.18	0.09	11	13		
	(37, 147)	(15, 174)	(0.08, 0.67)	(0.07, 0.70)	(10, 14)	(11, 16)		
***p***	0.853	0.591	0.010	0.179	0.069	0.614	0.085	0.761

CRP, c-reactive protein; WBC, white blood cells. Data are presented as medians variables are presented as medians (25th, 75th percentiles). The *p*-value refers to the results of Friedman’s ANOVA test, which analyzed the concentrations of parameters over time within each group. * indicates statistically significant difference between pregnant and nonpregnant patients at the specific time point (*p* ≤ 0.05).

**Table 5:** Clinical parameters in pregnant and nonpregnant patients recorded at four time points: T1 (the day of admission to the ICU), T2 (day 3 of ICU treatment), T3 (day 5 of ICU treatment), and T4 (day 7 of ICU treatment).

	D-Dimers, (mg/L)		Creatinine, (mg/dL)		Lactate, mmol/L		LDH, (U/L)	
Time	Pregnant	Nonpregnant	Pregnant	Nonpregnant	Pregnant	Nonpregnant	Pregnant	Nonpregnant
**T1**	1.8	3.8	0.5 *	0.8	1.3 *	2.1	479 *	840
	(1.6, 4.3)	(1.4, 17.4)	(0.4, 0.6)	(0.5, 1.9)	(1.1, 1.7)	(1.7, 3.7)	(333, 592)	(541, 1176)
**T2**	1.6	2.2	0.5	0.5	1.6	1.5	460	579
	(1.1, 2.2)	(1.3, 14.2)	(0.5, 0.7)	(0.5, 0.9)	(1.3, 1.9)	(1.2, 2.1)	(348, 583)	(345, 848)
**T3**	1.8	1.6	0.5	0.7	1.3	1.2	455	637
	(1.3, 3.6)	(1.1, 11.4)	(0.4, 0.6)	(0.5, 1.0)	(1.2, 1.7)	(0.9, 1.8)	(403, 526)	(339, 817)
**T4**	2.4	4.3	0.4	0.6	1.2	1.2		
	(1.4, 16.0)	(1.1, 17.1)	(0.4, 0.5)	(0.5, 0.9)	(1.1, 1.6)	(1.2, 2.1)		
***p***	0.221	0.831	0.384	0.289	0.272	0.001	0.513	0.090

LDH, lactate dehydrogenase. Variables are presented as medians (25th, 75th percentiles). The *p*-value refers to the results of Friedman’s ANOVA test, which analyzed the concentrations of parameters over time within each group. * indicates statistically significant difference between pregnant and nonpregnant patients at the specific time point (*p* ≤ 0.05).

**Table 6:** Pregnancy and neonatal outcomes.

Parameter	Pregnant, N = 14
**Number of pregnancies, N (%)**	
1st	2 (14)
2nd	6 (43)
3rd	2 (14)
4th	3 (21)
5th	1 (7)
**Length of pregnancy on ICU admission:**	
18–31 week	8 (57)
32–37 week	6 (43)
**Pregnancy outcomes, N (%):**	
Cesarean on the day of ICU admission	7 (50)
Cesarean during ICU stay	4 (29)
Pregnant, discharged home	3 (21)
**Neonatal outcomes, N (%):**	
Preterm birth during ICU stay	8 (58)
Full-term birth during ICU stay	3 (21)
Delivery after discharge from the hospital	3 (21)
**Neonatal survival, N (%)**	12 (86)

## Data Availability

The data set used and analyzed for the current study is available from the corresponding author upon reasonable request.
